# Current status and influential factors for family health management during quarantine: A latent category analysis

**DOI:** 10.1371/journal.pone.0265406

**Published:** 2022-04-21

**Authors:** Guangming Li, Mengying Li, Shuzhen Peng, Ying Wang, Li Ran, Xuyu Chen, Ling Zhang, Sirong Zhu, Qi Chen, Wenjing Wang, Yang Xu, Yubin Zhang, Xiaodong Tan

**Affiliations:** 1 Department of Preventive Medicine, School of Public Health, Wuhan University, Wuhan, Hubei, China; 2 Department of Health Management, The People’s Hospital of Huangpi, Wuhan, Hubei, China; 3 Department of Hospital Infection Management, Zhongnan Hospital of Wuhan University, Wuhan, Hubei, China; 4 Department of Geography, The College of Geography and Environment, Henan University, Kaifeng, Henan, China; 5 Department of Geography, National Earth System Science Data Center, National Science & Technology Infrastructure of China, Beijing, China; 6 Department of Health management, Wuchang Center for Disease Control and Prevention, Wuhan, Hubei, China; CNR, National Research Council of Italy, ITALY

## Abstract

**Objective:**

We aimed to explore factors affecting family health management during home quarantine as well as the effects of variations in family health management (FHM) on individuals’ health status.

**Methods:**

Using stratified random sampling, 618 families in Wuhan as well as cities within its surrounding provinces were recruited and surveyed online. Latent class variables were extracted from four modules: disinfection, space layout, physical exercise, and food reserves. The analysis was conducted using the *poLCA* package in R software (v.4.1.0). Chi-squared tests, Fisher’s exact tests, and non-parametric Kruskal–Wallis tests were used to compare groups as appropriate.

**Results:**

We found an overall questionnaire reliability of 0.77 and a total omega of 0.92, indicating that the survey results were credible. The Bayesian information criterion and Akaike information criterion were used to identified four latent class variables, namely latent non-family health management (18.9%) and latent low, medium, and advanced FHM (30.93%, 29.49%, and 20.59%, respectively). Gender, household income level, body mass index, the presence of a nearby community hospital, and self-rated health status showed statistically significant differences with respect to latent FHM. Moreover, we found a statistically significant difference in emotional reactions when comparing latent advanced and low to mid-level latent FHM. Compared with latent non-family health managers, we detected statistically significant differences in individual energy levels between potential family health managers at latent low and medium levels. Additionally, we found statistically significant differences in individual energy levels between latent advanced and low level family health managers.

**Conclusions:**

We found that multiple factors, including gender, household income, and body mass index, were correlated with latent FHM during home quarantine. We conclude that FHM can meaningfully improve individuals’ health. Thus, increasing social support for individuals can improve FHM as well as individuals’ health during home quarantine.

## Introduction

In response to the December 2019 outbreak of coronavirus disease 2019 (COVID-19) originating in Wuhan, China [1], the city was sealed off on January 23, 2020 in order to help to control the source of infection as well as the transmission of the virus [2,3]. Despite these efforts, subsequent outbreaks have since been observed in nearly every other country worldwide. On January 30, 2020, the World Health Organization (WHO) declared the novel coronavirus as a public health emergency of international concern [4,5]. As of July 17, 2021, 188 million cases and 406,700 deaths had been confirmed worldwide. Accordingly, COVID-19 has had an unprecedented direct impact on global health. However, the indirect effects of isolation policies on individual health must be explored, especially from the perspective of family health [6].

The family management framework was originally proposed to assess the influence of family on chronic disease management and recovery [7], demonstrating that individualized family health management (FHM) can help patients manage their disease, recover their health, and improve their quality of life [8]. FHM has become increasingly recognized as an integral component of chronic disease management and a means of secondary prevention, thus helping reduce the burden of chronic diseases on individuals, families, and communities [9]. Recently, Knafl et al. [10] reported an expansion of the family management framework and found that family social networks, health care, education, and access to various resources were also impactful [10]. Moreover, the authors showed positive impacts of FHM on children’s health and family relations [11]. Nomi et al. [12] again redefined the concept of family health, pointing out that, as a “resource at the level of the family unit that develops from the intersection of the health of each family member, their interactions and capacities, as well as the family’s physical, social, emotional, economic, and medical resources…family health is greater than the sum of its parts.” Optimal family health promotes a sense of belonging and a capacity to develop and adapt, to care for others, and to meet interpersonal responsibilities. Accordingly, family health is one of the most effective and powerful ways to either develop, promote, or degrade individuals’ health [12].

Previous studies have found that, in the absence of a vaccine, a public health policy promoting non-pharmaceutical interventions, close contact tracing, and the isolation of confirmed cases can help reduce the spread of the disease [13,14]. As a result, numerous countries have issued household orders and adopted social distance isolation policies over the course of the past two years [15]. These measures have included closing factories and schools, suspending non-essential commerce and trade, educating individuals to maintain a ≥2 m distance from others [16], and mandated home isolation [17,18] (i.e., wherein residents are forced to work, study, and reside in their homes for 10–24 h per day).

Locally, the government of Wuhan implemented a strict home isolation policy that lasted for 76 days [19]. Due to the high risk of community transmission, neighboring provinces and cities implemented the same policy [20]. During this home quarantine period, residents stayed in their homes for up to 24 h per day, with severely restricted forms of outside activity. Thus, the pre-epidemic concept of a family home was revolutionized for the purpose of resisting viral infection [21], as home was no longer simply a place to eat and sleep, but instead became the center of study, work, exercise, and social life. These alterations have resulted in changes to family structure and the home environment on a global scale. Accordingly, how to best promote healthy family life and protect individuals’ health from an FHM perspective during the COVID-19 pandemic has become an extremely important topic of concern to many researchers, policymakers, and healthcare workers. Although multiple studies have focused on individuals’ mental health status, prior research efforts have been notably sparse and have likewise neglected the influence of family functionality and structuring on family health. Moreover, to our knowledge, prior studies have not explored the influencing factors that are relevant to FHM during home isolation [22–25].

Accordingly, due to the present lack of research on individual health from a family health perspective, the current study adopted a latent category analysis method to explore the impact of FHM style and to analyze the influential factors relevant to FHM patterns as well as their impacts on individual health. We also aimed to provide a reference for optimal FHM for the purpose of addressing the current pandemic as well as future public health emergencies.

## Methods

### Survey

The WHO cites provincial- and city-level virus traceability survey results from China [26] and Johns Hopkins University as important resources within big epidemic data with regard to informing public health preparedness and the pandemic response [27], and the current survey was designed based on data arising from these research efforts.

**Fig 1:**
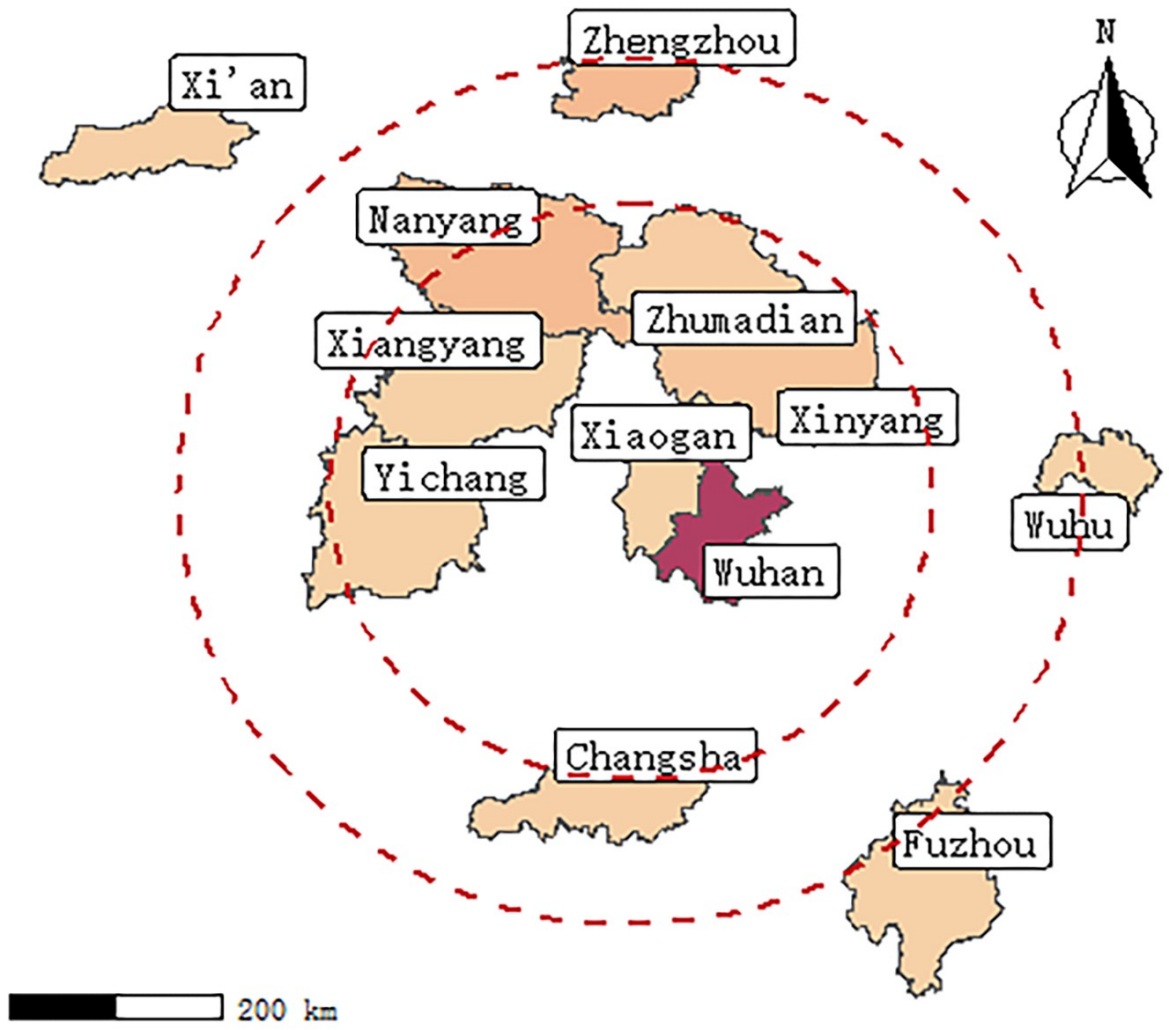
Range of research subjects selected for the current study.

The Hubei province was partitioned by geographic distance and the number of confirmed cases, and the participants of the current study were recruited via random sampling of the surrounding provinces and cities. This study procedure yielded data for the following locations: Xinyang, Zhumadian, Nanyang, and Zhengzhou City in Henan province; Xiaogan, Xiangyang, Yichang, and Wuhan in Hubei Province; Xi’an in Shanxi Province; Fuzhou in Jiangxi Province; Changsha in Hunan Province; and Wuhu in Anhui Province (Fig 1).

A stratified random sampling method was adopted to randomly code the selected cities by street, community, and family unit, with a certain proportion of subjects selected from each respective category. The final families selected for inclusion in the current study were taken as the investigation objects using the following criteria for the selection of survey subjects: familiarity with the life and environment of all family members, living with the family for ≥3 months during the period of home isolation (from January 23 to April 8, 2021), and adults ≥18 years of age.

#### Survey method

Using family contact information provided by the community, we randomly selected survey objects that met the aforementioned inclusion criteria. Following respondent selection, a survey description was distributed to convey the study’s intention. The online questionnaire was distributed using the Questionnaire Star platform (https://www.questionstar.com/).

#### Investigative content

The survey content included three primary components: basic population characteristics (including age, gender, place of residence during the pandemic, monthly family income, and various other indicators); the FHM module, which was divided into four aspects (household disinfection, transformation of family space, exercise, food reserves); and the Nottingham Health Scale, which was used to evaluate individual health status [28,29] and consisted of 38 items across six primary dimensions (functionality level, pain, emotional response, sleep, social isolation, physical ability). All dimensions were mutually independent and had a possible score of 100; the higher the original score, the worse the physical condition (and vice versa).

### Statistical analysis

City-level results were combined from within the collected questionnaires. Invalid and spurious questionnaires (i.e., those not meeting study inclusion criteria due to excessive missingness or not completing the study questionnaire) were eliminated, whereas missing values within valid questionnaire responses were processed via mean interpolation. The scores of each dimension were summed according to the Nottingham Health Scale using the *psych* package in R (RStudio *v*.1.4.1106; R *v*.4.1.0; The R Project for Statistical Computing, Vienna, Austria) to perform reliability tests.

We identified the following statistically significant FHM variables: family food storage, the presence of a disinfection space, disinfection equipment, an identified disinfection solution, whether the family space was reconstructed, and whether the family exercised. Dummy variables for family food reserves and physical exercise were processed. The former variable (insufficient family food reserves) was divided into four levels, three of which were generated by taking family food sufficiency as the reference variable. Alternatively, family exercise was divided into five levels, comprising a no exercise referent and four dummy variables.

The latent FHM categories were determined according to the following parameters: the Akaike information criterion (AIC), the Bayesian information criterion (BIC), adjusted the Bayesian information criterion (aBIC), and relative entropy. The *poLCA*, *PMCMRPlus*, *Gmodels*, and *ggplot2* R packages were implemented for analyzing factors affecting latent FHM as well as exploring their effects on individual health [30]. Qualitative data were expressed as percentages. Pairwise comparisons were performed via chi-squared or non-parametric rank sum tests and intergroup comparisons were performed via the Kruskal–Wallis test. The Bonferroni method was used to correct for pairwise comparisons. All statistical significance levels were set to an *α* of *≤*0.05.

### Ethics statement

As this study was conducted during the course of COVID-19 prevention and control practice efforts within public health units and the online survey was a supplementary survey that was accessed for a retrospective investigation of epidemiological data, ethics approval was formally waived for this secondary analysis by the ethics review board at our institution. However, the original investigation was reviewed by our affiliated ethics review board and we obtained subjects’ verbal informed consent before initiating the questionnaire. This study was conducted in accordance with the principles of the Declaration of Helsinki and its later amendments.

### Patient and public involvement

No patient was involved in the study design, in determining the research questions, in interpreting or writing up the study results, or in otherwise reporting this research, nor was any entity within the public at large.

## Results

### Questionnaire reliability and validity

A total of 710 questionnaires were collected, 92 of which were excluded based on the integrity of the reverse questions and questionnaire responses, thus yielding a total of 618 valid questionnaires (effective response rate, 87.04%). The resulting KMO (Kaiser-Meyer-Olkin) value was 0.76 and Bartlett’s sphericity test yielded a value of 492.42 (p = 2*10^−16^). The total questionnaire reliability was 0.77 and the split-half reliability was 0.78. The average R and median values of the project were notably similar (0.36 and 0.35, respectively). The hierarchical, progressive, and total omega values (0.71, 0.77, and 0.92) further indicated the reliability of the survey results (Table 1).

**Table 1:** Questionnaire reliability and validity analysis.

	Mean±SD	Std.alpha	G6(smc)	Average_r	Med.r
Energy level	19.75±31.84	0.72	0.72	0.34	0.35
Pain	9.38±20.18	0.76	0.75	0.39	0.36
Emotional reaction	16.15±23.98	0.70	0.70	0.32	0.35
Sleep	39.72±40.66	0.76	0.75	0.38	0.36
Social isolation	34.08±38.80	0.77	0.77	0.40	0.40
Physical abilities	17.92±24.12	0.73	0.74	0.35	0.32

Note: SD, standard deviation; Std.alpha, standarized alpha based upon the correlations; G6(smc), guttman’s lambda 6 reliability; Average_r, average interitem correlation; Med.r, median interitem correlation.

### Latent category analysis

**Fig 2:**
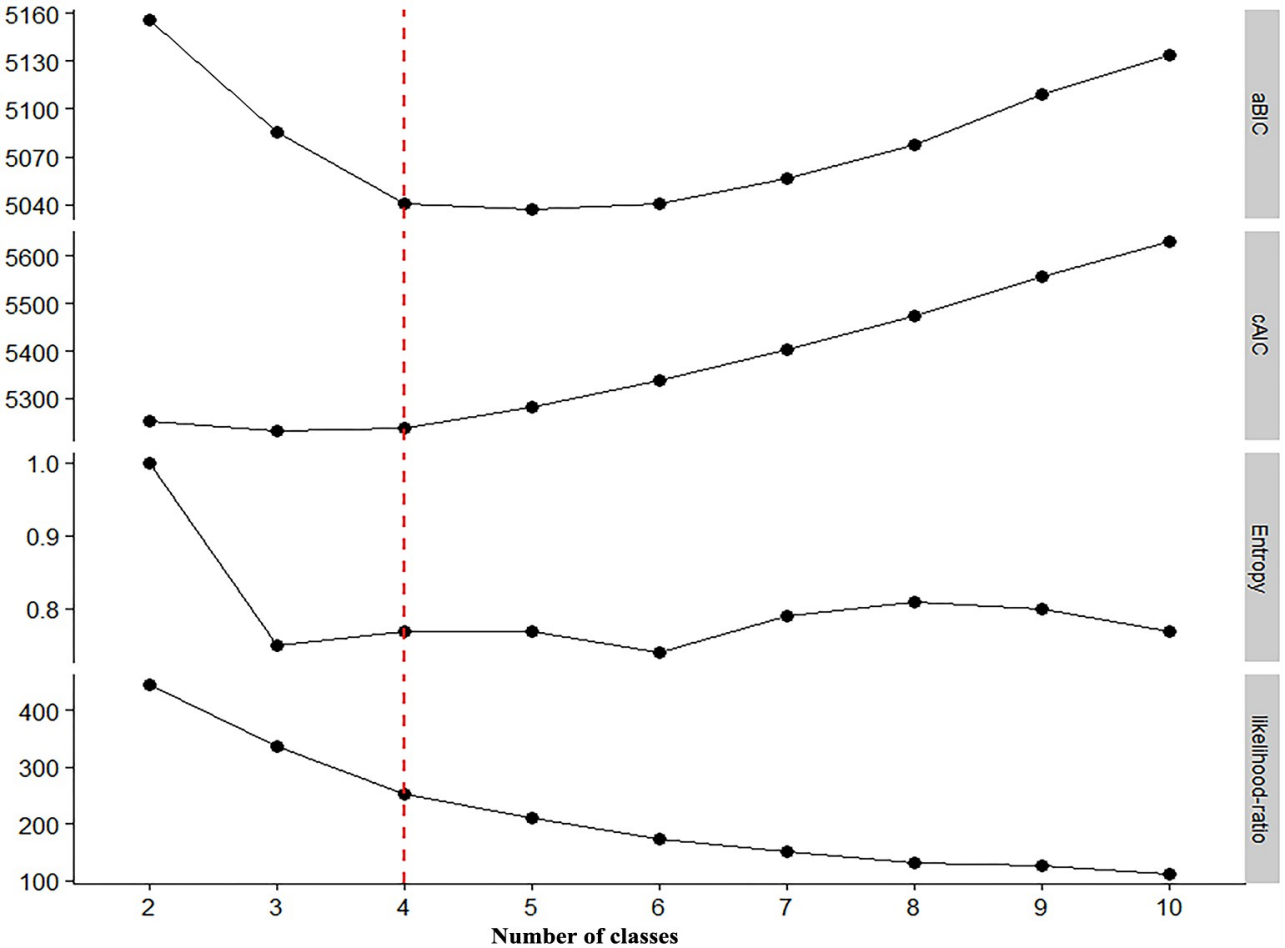
Number of latent categories screened via information criteria.

**Fig 3:**
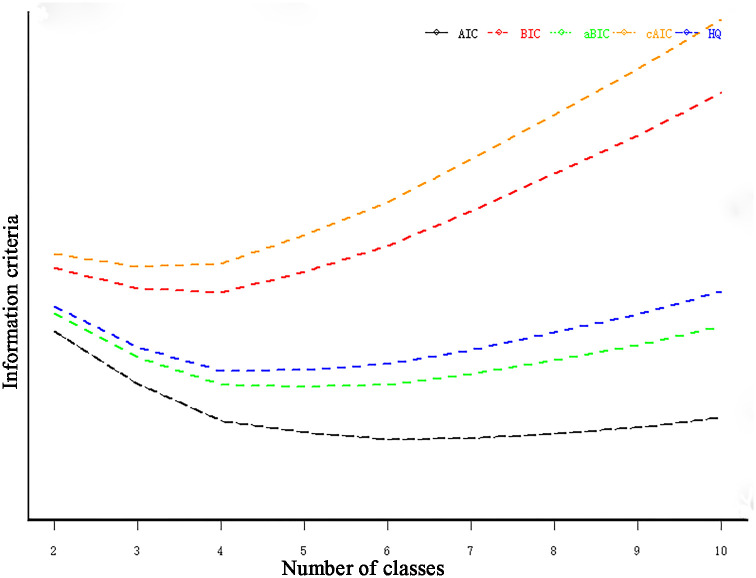
Comparison of information criteria for selecting the number of classes.

**Fig 4:**
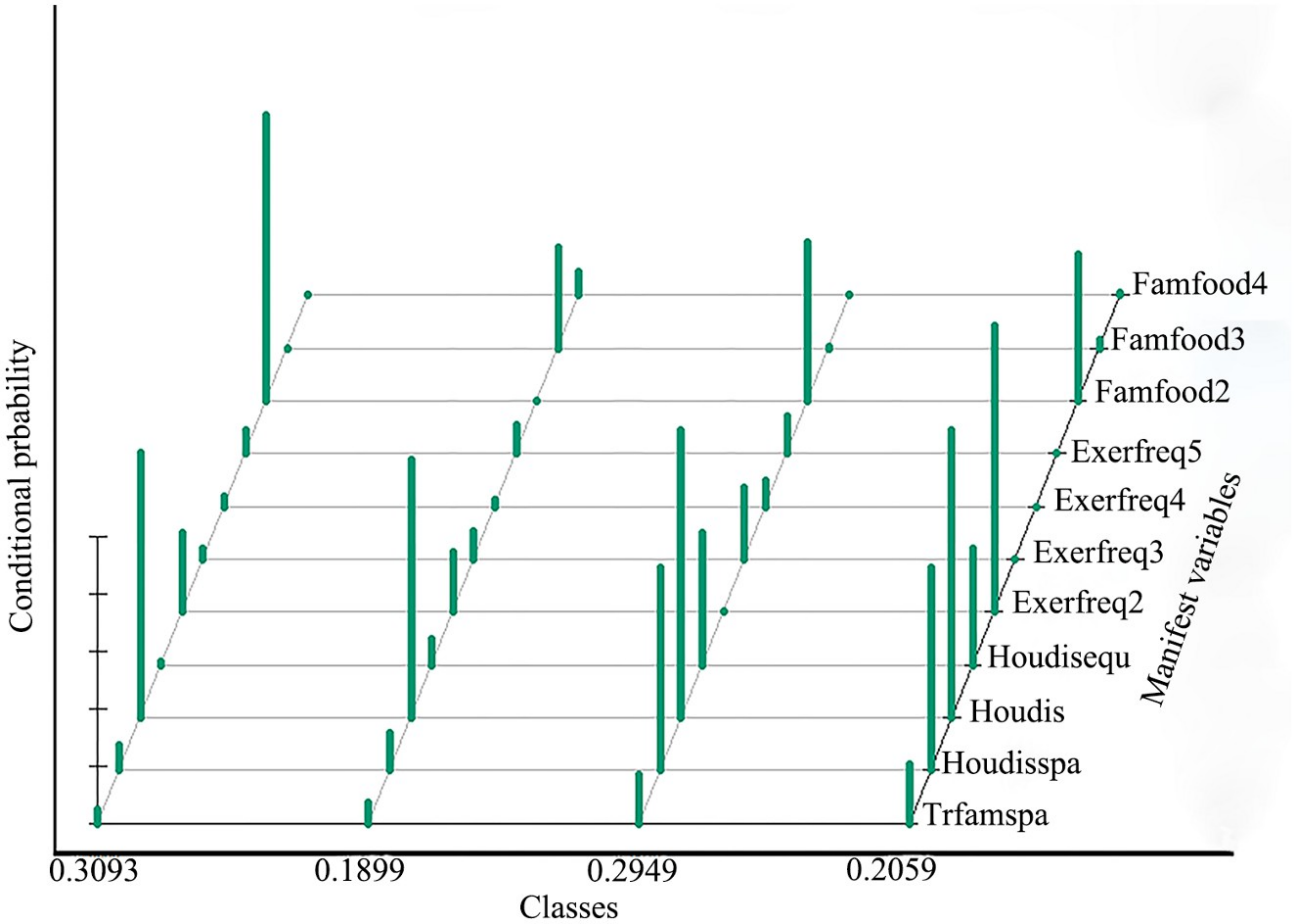
Posterior probability of manifest variable responses across each class. **Notes**: Trfamspa, transformation of family space; Houdisspa, household disinfection space; Houdisequ, household disinfection equipment; Houdis, household disinfection; Exerfreq2, exercise frequency (1~2 sessions/week); Exerfreq3, exercise frequency (3~4 sessions/week); Exerfreq4, exercise frequency (5~6 sessions/week); Exerfreq5, exercise frequency (one sessions/day); Famfood2, rationing specific basic supplies; Famfood3, family food shortage; Famfood4, no family food reserves.

When the number of latent classes was set to four, the cAIC was 5237, the aBIC was 5041, the relative entropy was 0.77, and the maximum likelihood ratio was 252 (where a relative entropy of >0.7 indicates an acceptable classification accuracy; see Fig 2, dotted line). When the latent category index was set to five, the aBIC, modified AIC (mAIC), modified version of the AIC (AIC3) [31], and Hurvich and Tsai criterion were at their minimum values; whereas BIC and cAIC reached their minimum values when the latent category index was set to four (Table 2). Regarding AIC, BIC, and aBIC, the smaller the values, the better the model fit. According to the observed situation, the degree of interpretation, and the graphed inflection points of the latent categories (the data was ultimately divided into four latent categories, Fig 3), the overall probabilities for each latent category were as follows: Class 1, 0.3093; Class 2, 0.1899; Class 3, 0.2949; and Class 4, 0.2059 (Fig 4).

**Table 2:** Information criteria for the selected classification variables.

Df	Gsq	Llink	AIC	mAIC	AICc	HT	cAIC	AIC3	BIC	aBIC	HQ	Nclass
595	446	-2541	5128	5151	5130	5130	5253	5154	5230	5157	5168	2
583	335.8	-2486	5042	5077	5046	5046	5232	5083	5197	5086	5102	3
571	252	-2444	4982	5029	4990	4990	5237	5040	5190	5041	5063	4
559	209.4	-2423	4963	5022	4976	4977	5284	5039	5225	5037	5065	5
547	173.6	-2405	4952	5023	4970	4971	5337	5047	5266	5040	5074	6
535	152.4	-2394	4954	5037	4980	4981	5405	5070	5322	5058	5097	7
523	136.4	-2386	4962	5057	4997	4998	5478	5101	5383	5081	5126	8
511	120.9	-2378	4971	5078	5016	5017	5552	5135	5445	5105	5155	9
499	112.8	-2374	4987	5106	5044	5045	5633	5177	5514	5136	5192	10

**Notes**: Df, degree of freedom; Gsq, likelihood ratio/deviance statistic; HQ, Hurvich and Tsai criterion; AICc, corrected Akaike information criterion; Nclass, number of classes.

### Latent class characteristics and nomenclature

**Fig 5:**
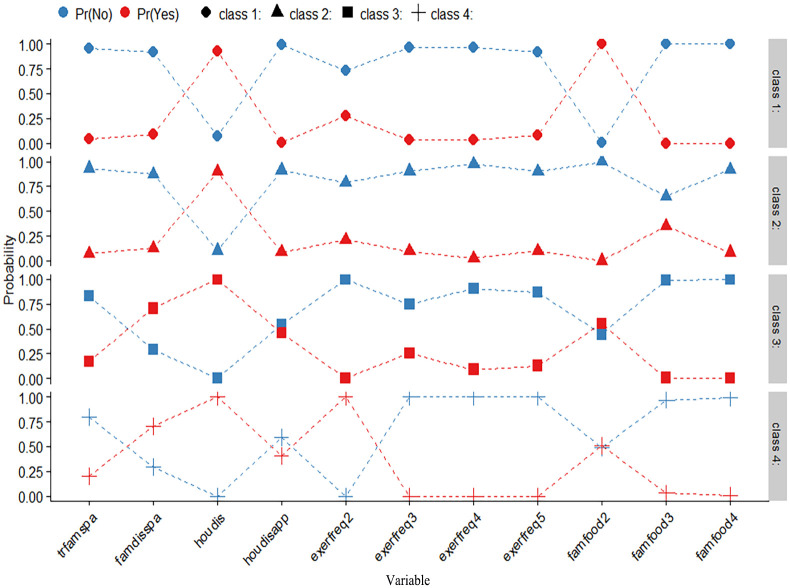
Conditional probability distributions of manifest variable responses by class.

The conditional probabilities for each index are shown in Fig 5. In the first category, the conditional probabilities were found to be low overall, with eight indices of <10% and three indices of approximately 20%. With respect to food and household disinfection, the indices reflecting basic sufficiency were 92.7% and 100%, respectively, whereas the corresponding index for 1–2 exercise sessions per week was 27.2%. For the second category, we noted six indicators of <10% and three indicators of >20%, with conditional probabilities for household disinfection, family food shortages, and 1–2 sessions of weekly exercise of 89.8%, 35.0%, and 21.0%, respectively. Alternatively, the third category included information on household disinfection equipment and 3–4 exercise sessions per week, with resulting conditional probabilities of 45.8% and 25.2%, respectively, and a conditional probability of family food storage sufficiency of 55.4%. In the fourth category, we noted six indicators of >20%; the conditional probability for the family space renovation indicator was 20.2%, the conditional probabilities for family disinfectant use and 1–2 sessions of weekly exercise were each 100%, and the probabilities for the presence of a household disinfection space, disinfection equipment, and food saving practices were reasonably sufficient (70.6%, 40.5%, and 51.1%, respectively). According to the overall level of conditional probabilities for the different latent categories and indicator characteristics evaluated herein, we adopted a method of combining information on degree and feature naming. As the conditional probability varied from small to large, the following four categories were named: latent non-FHM (Class 1), and latent low, medium, and advanced FHM (Classes 2–4).

### Influential factors for latent FHM

Overall, gender, family income level, body mass index (BMI), the presence of a nearby community hospital, and self-rated health status had the most statistically significant effects on latent FHM. This study enrolled 175 males (28.3%) and 443 females (71.7%). We found statistically significant differences in latent FHM categories between genders (p = 0.04). For BMI, 53 (8.6%) individuals were found to be underweight, 385 (62.3%) were of average BMI, 143 (23.1%) were overweight, and 37 (6.0%) were obese, resulting in statistically significant differences in latent FHM across BMI categories (p = 0.0339). When evaluating household income variables, 92 families (14.9%) were in the lowest income bracket, 259 (41.9%) were considered low income, 174 (28.2%) were considered middle income, and 93 (15.0%) were deemed high income families, again with statistically significant differences observed between income categories (p = 0.023). Among the respondents, 563 (91.1%) lived near community hospitals, while 55 (8.9%) did not. The effects of this difference on latent FHM patterns was statistically significant (p = 0.039). In total, 346 (56.0%) individuals were found to be completely healthy (i.e., with no chronic diseases), 256 (41.4%) were categorized into the “sub-health” level (i.e., no chronic diseases, but with lower level health concerns such as insomnia or psychological stress), and 16 (2.6%) had some level of chronic disease based on self-reported data, with statistically significant differences in latent FHM categories observed when comparing across health status groups (p = 0.00016). Age, occupation, place of residence, education, marital status, urban and rural health insurance, commercial health insurance, confirmed cases of novel coronavirus in the vicinity, and the presence of remote students or workers in the family unit each displayed no statistically significant differences with regard to latent FHM (Table 3).

**Table 3:** Influential factors for latent family health management (FHM).

	Freq.(n (%))	Non-family health management (NFHM)	Low-level family health management (LFHM)	Medium-level family health management (MFHM)	Advanced family health management (AFHM)	*p*
		44 (40.3%)	249 (7.1%)	264 (42.7%)	61 (9.9%)	
Gender:						0.04
Male	175(28.3%)	15 (34.1%)	79 (31.7%)	72 (27.3%)	9 (14.8%)	
Female	443(71.7%)	29 (65.9%)	170 (68.3%)	192 (72.7%)	52 (85.2%)	
Age						0.327
Youth (18–40 years)	370(59.9%)	28 (63.6%)	157 (63.1%)	156 (59.1%)	29 (47.5%)	
Middle age (41–65 years)	246(39.8%)	16 (36.4%)	91 (36.5%)	107 (40.5%)	32 (52.5%)	
Elderly (≥66 years)	2(0.3%)	0 (0.00%)	1 (0.40%)	1 (0.38%)	0 (0.00%)	
Occupation						0.562
Non-medical staff	288(46.6%)	20 (45.5%)	110 (44.2%)	125 (47.3%)	33 (54.1%)	
Medical staff	330(53.4%)	24 (54.5%)	139 (55.8%)	139 (52.7%)	28 (45.9%)	
Body mass index (BMI)						0.0339
Underweight (BMI<18.5)	53(8.6%)	5 (11.4%)	23 (9.24%)	21 (7.95%)	4 (6.56%)	
Normal weight (18.5≤BMI<24.0)	385(62.3%)	22 (50.0%)	145 (58.2%)	172 (65.2%)	46 (75.4%)	
Overweight (24.0≤BMI<28.0)	143(23.1%)	16 (36.4%)	57 (22.9%)	60 (22.7%)	10 (16.4%)	
Obese (BMI≥28)	37(6.0%)	1 (2.27%)	24 (9.64%)	11 (4.17%)	1 (1.64%)	
Place of residence						0.935
Rural	134(21.7%)	11 (25.0%)	55 (22.1%)	55 (20.8%)	13 (21.3%)	
City	484(78.3%)	33 (75.0%)	194 (77.9%)	209 (79.2%)	48 (78.7%)	
Education						0.4
Junior high school and below	12(1.9%)	1 (2.27%)	3 (1.20%)	6 (2.27%)	2 (3.28%)	
High school or technical school	67(10.8%)	8 (18.2%)	30 (12.0%)	22 (8.33%)	7 (11.5%)	
Undergraduate / college	439(71.0%)	31 (70.5%)	179 (71.9%)	190 (72.0%)	39 (63.9%)	
Master’s degree	84(13.6%)	3 (6.82%)	33 (13.3%)	38 (14.4%)	10 (16.4%)	
Doctoral degree and above	16(2.6%)	1 (2.27%)	4 (1.61%)	8 (3.03%)	3 (4.92%)	
Marriage						0.2151
Unmarried	164(26.5%)	13 (29.5%)	58 (23.3%)	74 (28.0%)	19 (31.1%)	
Married	435(70.4%)	29 (65.9%)	181 (72.7%)	183 (69.3%)	42 (68.9%)	
Divorce	16(2.6%)	1 (2.27%)	10 (4.02%)	5 (1.89%)	0 (0.00%)	
Widowed	2(0.3%)	0 (0.00%)	0 (0.00%)	2 (0.76%)	0 (0.00%)	
Other	1(0.2%)	1 (2.27%)	0 (0.00%)	0 (0.00%)	0 (0.00%)	
Family income						0.023
Lowest (<2,000 ¥/month)	92(14.9%)	10 (22.7%)	31 (12.4%)	39 (14.8%)	12 (19.7%)	
Low (2,001–5,000 ¥/month)	259(41.9%)	20 (45.5%)	101 (40.6%)	123 (46.6%)	15 (24.6%)	
Middle (5,001–10,000 ¥/month)	174(28.2%)	11 (25.0%)	69 (27.7%)	71 (26.9%)	23 (37.7%)	
High (>10.000 ¥/month)	93(15.0%)	3 (6.82%)	48 (19.3%)	31 (11.7%)	11 (18.0%)	
Urban/rural health insurance						0.750
No	80(12.9%)	6 (13.6%)	30 (12.0%)	38 (14.4%)	6 (9.84%)	
Yes	538(87.1%)	38 (86.4%)	219 (88.0%)	226 (85.6%)	55 (90.2%)	
Commercial health insurance						0.086
No	385(62.3%)	32 (72.7%)	148 (59.4%)	173 (65.5%)	32 (52.5%)	
Yes	233(37.7%)	12 (27.3%)	101 (40.6%)	91 (34.5%)	29 (47.5%)	
COVID case in the vicinity						0.1265.
Yes	223(36.1%)	16 (36.4%)	96 (38.6%)	93 (35.2%)	18 (29.5%)	
No	330(53.4%)	20 (45.5%)	126 (50.6%)	143 (54.2%)	41 (67.2%)	
Unknown	65(10.5%)	8 (18.2%)	27 (10.8%)	28 (10.6%)	2 (3.28%)	
Nearby community hospitals						0.039
No	55(8.9%)	9 (20.5%)	16 (6.43%)	25 (9.47%)	5 (8.20%)	
Yes	563(91.1%)	35 (79.5%)	233 (93.6%)	239 (90.5%)	56 (91.8%)	
Remote student/worker in family						0.058
No	148(23.9%)	12 (27.3%)	63 (25.3%)	67 (25.4%)	6 (9.84%)	
Yes	470(76.1%)	32 (72.7%)	186 (74.7%)	197 (74.6%)	55 (90.2%)	
Self-rated health status						0.00016
Completely healthy	346(56.0%)	23 (52.3%)	160 (64.3%)	119 (45.1%)	44 (72.1%)	
Sub-health (non-chronic)	256(41.4%)	20 (45.5%)	84 (33.7%)	136 (51.5%)	16 (26.2%)	
Chronic disease	16(2.6%)	1 (2.27%)	5 (2.01%)	9 (3.41%)	1 (1.64%)	

### Latent FHM modes and individual health status

**Fig 6:**
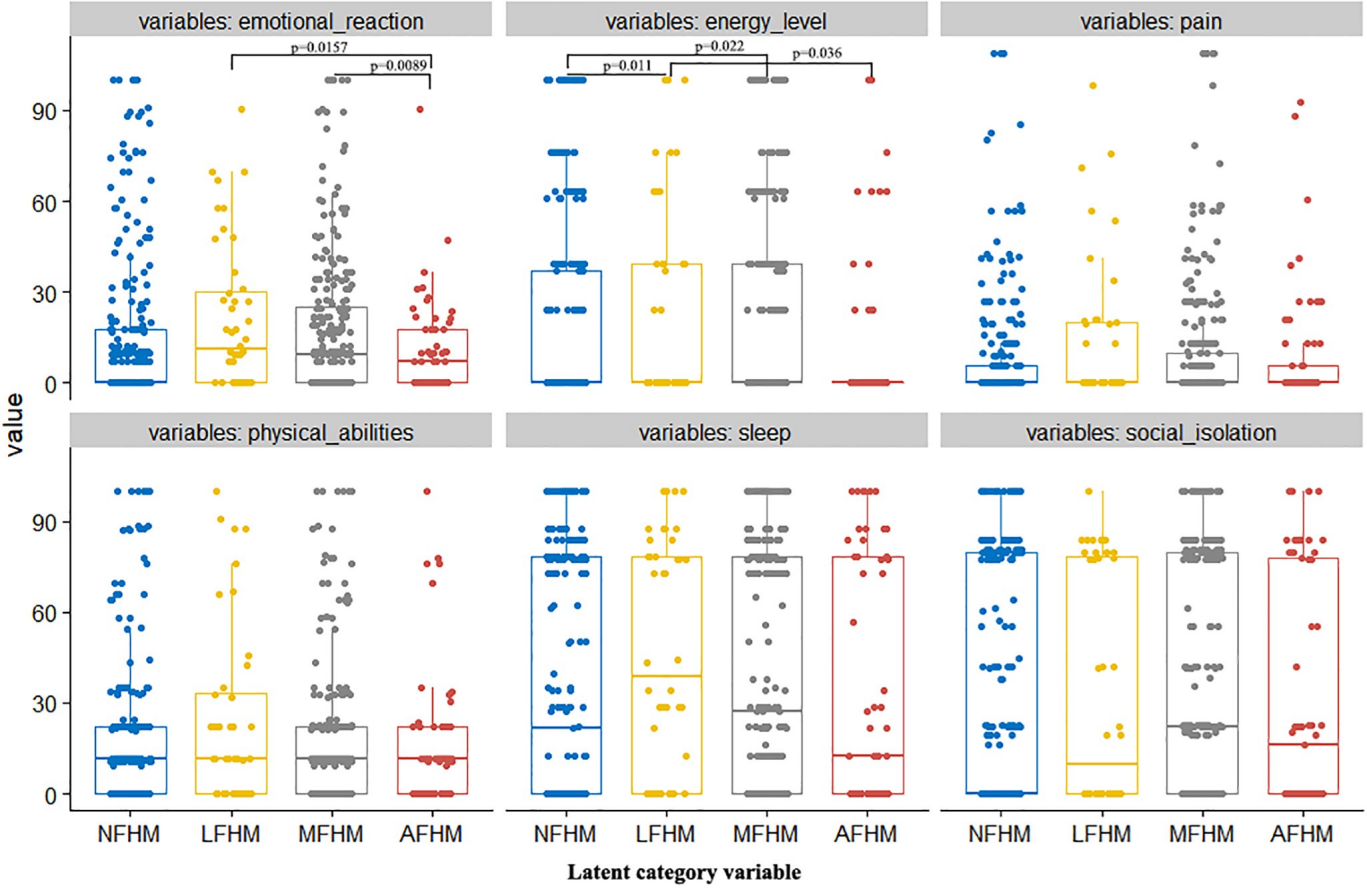
Effects of different latent FHM (family health management) modes on individual health status.

We found statistically significant differences in emotional reactions between latent advanced vs. low- and mid-level latent FHM approaches (both p = 0.0157 and p = 0.0089). Compared with non-family health managers, we found statistically significant differences in individual energy levels between low-level and medium-level family health managers (both p = 0.011 and p = 0.022). Moreover, there were statistically significant differences in individual energy between advanced and low-level family health managers (p = 0.036; Fig 6). There were no statistically significant differences observed with regard to pain, physical activity, sleep, and social isolation among the different FHM modes.

## Discussion

The present study is significant in that, in contrast to previous FHM analyses that have focused primarily on home care or health management of a single family member with a serious or chronic illness [32,33], the research objective herein was to evaluate healthy, normal individuals within the family unit under conditions of home isolation following the emergence of a highly infectious disease. This study found that, in the case of home isolation occurring during a major infectious disease event, most families were highly capable of carrying out effective FHM. Moreover, we found that FHM can play a positive role in promoting improvements in individual health status. Accordingly, this study can serve as a reference to help family members maintain their health during home isolation during this outbreak as well as any similar future outbreaks.

Our findings demonstrate that, within this study population, family income level had a statistically significant impact on latent FHM patterns. Namely, the greater the family income, the more feasible it was to carry out higher levels of latent FHM. Wu et al. [34] found that family income can positively affect the health status of children by meaningfully improving material living conditions. A study by Conger et al. [35] similarly concluded that family socioeconomic status plays an important role in family life support and personal development. Accordingly, it is fitting that family income may play a role in promoting FHM. In addition, the current study demonstrated that women maintain higher levels of latent FHM. As women typically do more housework than men in their domestic lives (i.e., women typically take on up to two-thirds of the necessary housework [36]) and spend nearly twice as much time at home working and caring for children as compared with men [37], their investment in latent FHM may be correspondingly higher.

Additionally, we found that BMI can influence FHM and vice versa. Here, it was found that the majority of people with a BMI of 18.5–24 kg·m^-2^ (i.e., those with normal weight for Asian populations) undertook FHM, accounting for 58.2% of low-level, 65.2% of mid-level, and 75.4% of advanced FHM. Similarly, studies conducted in China and the US have demonstrated a statistically significant non-linear relationship between BMI and health-related quality of life [38,39]. A separate US study demonstrated that women had the highest health-related quality of life when their BMI was 22 kg·m^-2^, whereas the same was true for males with a BMI of 22–30 kg·m^-2^ [40]. Beer [41] found that the health-related quality of life of obese adolescents decreased with increasing BMI. Moreover, Janet et al. [42] also found that FHM can statistically significantly improve health-related quality of life, demonstrate that the treatment of childhood obesity with a whole family participation model was statistically significantly correlated with improvements in child and adult BMI across the whole family unit [43]. Accordingly, we conclude that BMI can affect FHM by altering health-related quality of life, and this effect can be countered by effective FHM addressing concerns of overweight or obesity.

Moreover, our results showed that families with community hospitals nearby their homes were more likely to engage in higher levels of FHM. Since 2009, the primary goals of China’s medical reform efforts have been to strengthen the primary health care system [44], including gradually building a health management mode supported by family doctors and improving the level of FHM on a population scale. Accordingly, the visit rates of family doctors have gradually increased in different regions, essentially establishing a hierarchical diagnostic and treatment mechanism with respect to primary health care delivery [45]. In some regions in China, family doctors have achieved high signing rates (i.e., referring a program in China in which specific physicians may be selected and “signed” by individuals and families), and significant progress has been made toward achieving the national target of having a hospital within a 15 min driving distance for all citizens and residents [46,47]. The influence of family doctors on FHM may be roughly represented by the proximity of community hospitals. However, the influence of specific community hospitals and various associated factors on latent FHM still requires further exploration.

Increasing levels of family health may improve individuals’ levels of mental health as well. One study found that home isolation significantly reduced the opportunity for physical exercise in the elderly, thus affecting both their physical and mental health both directly and synergistically [48]. Additional studies have found correlations between isolation, anxiety, and loneliness in young children [49], with isolation leading to an approximately 4.7–10.3% increase in behavioral problems in school-age children throughout China [50]. These findings highlight the detrimental impact of isolation on mental health on a population scale [51]. For example, in Wuhan, 20% of students in home isolation showed symptoms of anxiety and depression [52,53]. Alternatively, our study revealed that a higher degree of latent FHM can improve psychological responses, thus improving individuals’ mental health during home isolation. These findings build upon the known physical health benefits of FHM discussed above. Moreover, our study found that improving FHM levels can significantly improve individuals’ energy levels.

Several limitations of our study should be considered: First, causality cannot be confirmed, as we only explored only correlations between latent FHM and influencing factors and did not conduct a prospective investigation that would allow for drawing causal inferences. Second, we did not conduct a latent category analysis based on the classical three-step method [54]. Fortunately, the comparative analyses based on the *poLCA* and *Mplus* packages as well as the generalized structured component analysis conducted herein yielded consistent results [55,56].

## Conclusions

In this study, multiple factors, including gender, family income, and BMI, were associated with latent FHM levels during home quarantine. Moreover, our findings support the contention that FHM should be an important focus for isolated families during epidemic and pandemic periods, as effective FHM can meaningfully improve individuals’ health. FHM may be promoted through increasing residential income, generating policy support, and conducting health education interventions on a population scale as well as from a preventive medicine perspective in order to improve FHM levels during pandemic conditions and overall. Our findings thus guide future research directions and inform policy decisions and medical guidelines.

## Supporting information

S1 Data (ZIP)

S1 File (PDF)
